# The Life and Work of Nikolai Ivanovich Pirogov (1810-1881): An Outstanding Anatomist and Surgeon

**DOI:** 10.7759/cureus.3424

**Published:** 2018-10-08

**Authors:** Konstantinos Koutsouflianiotis, George K Paraskevas, Eleni Zagelidou, Katerina Dimakopoulou, George Noussios

**Affiliations:** 1 Internal Medicine, General Hospital of Thessaloniki "G. Gennimatas", Thessaloniki, GRC; 2 Orthopaedics, Aristotle University of Thessaloniki, Thessaloniki, GRC; 3 Pathology, Aristotle University of Thessaloniki, Thessaloniki, GRC; 4 Otolaryngology, Aristotle University of Thessaloniki, Thessalonki, GRC

**Keywords:** anatomy, history, medicine, surgery, pirogov

## Abstract

Nikolai Ivanovich Pirogov is considered one of the most important anatomists and surgeons in the history of medicine. The Russian physician conducted more than 11,000 dissections and meticulously studied human anatomy, discovering important anatomical regions such as Pirogov's triangle. Pirogov developed surgical methods and techniques used by physicians for many decades such as Pirogov's amputation. Pirogov is also known for his contribution to war medicine, given his experience practicing medicine in the Crimean War as a surgeon, where he introduced innovative methods for the treatment of injured soldiers. Pirogov’s most important contribution to the scientific community is his humanistic and democratic mentality—which he maintained until the end of his life—elements necessary for the evolution of every modern physician and scientist.

## Introduction and background

**Figure 1:**
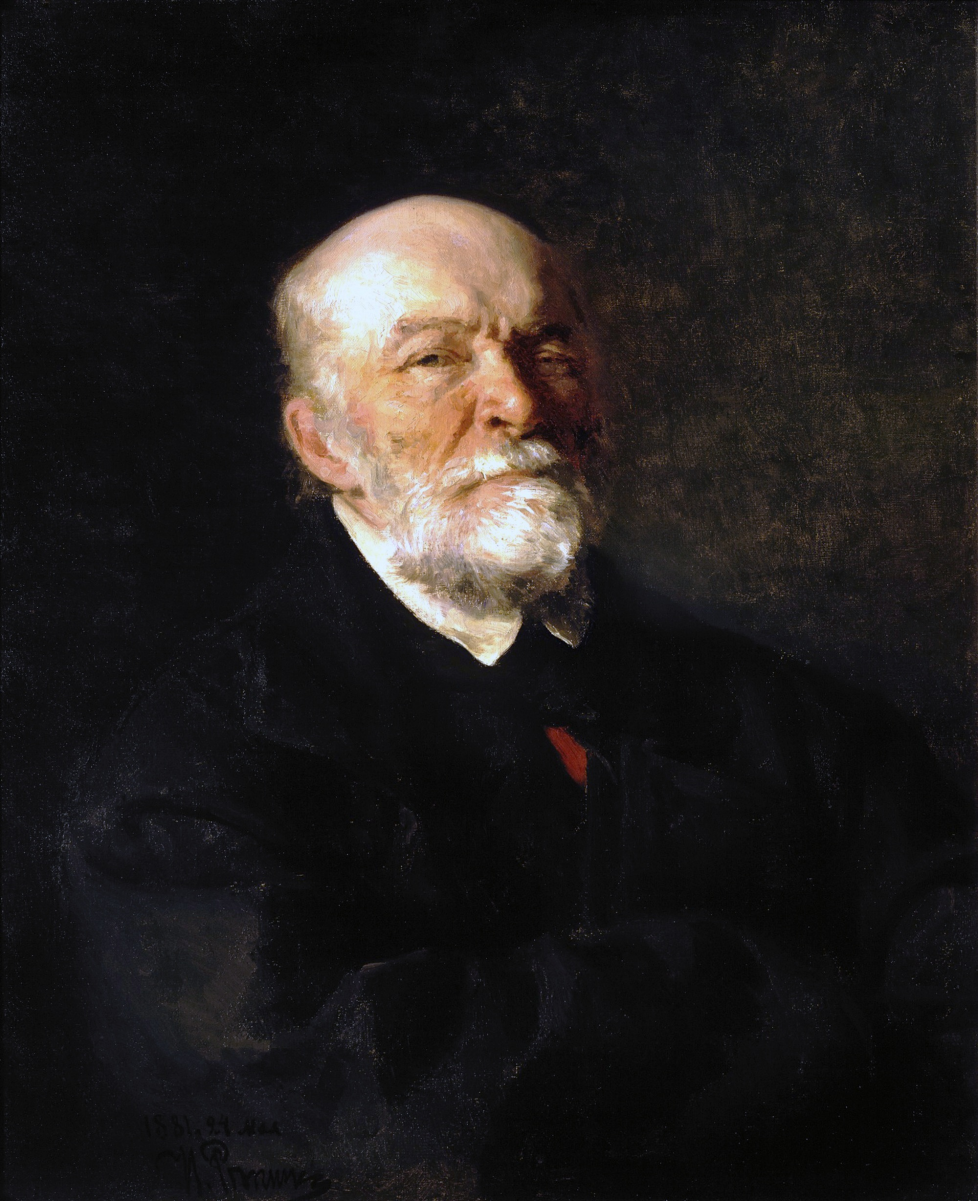
Nikolai Ivanovich Pirogov, portrait by Ilya Repin. Public Domain

Nikolai Ivanovich Pirogov (1810–1881) is considered an outstanding anatomist and surgeon originating from Russia, with a significant body of scientific work (Figure 1).

Although Pirogov contributed to the scientific community with important discoveries in anatomy, surgery and war medicine, he is not notably known among Western physicians. This study aims to highlight the life and work of Nikolai Pirogov for interested readers to gain knowledge and inspiration—two assets necessary for scientific evolution.

## Review

Pirogov was born in Moscow on November 13, 1810, and was the 13th child of a middle-class family. After the death of his father, Pirogov’s family encountered serious financial difficulties [1]. Nikolai Ivanovich, with the support of Professor Mukhin—a family friend and professor of anatomy and physiology at Medico-Surgical Academy and later at Moscow University—entered Moscow University as a medical student [2]. Pirogov passed the examination and was admitted to Moscow University at the age of 14, although the lowest age limit for entrance was 16. In his application for the entry exams, Pirogov stated that he was sixteen [3].

Pirogov’s university years were very difficult for the young student. His family’s financial difficulties and his remarkably young age made things even harder. The university was located far from his home, and he had to walk for hours every day. In winter, the trek was more difficult [4]. Furthermore, the educational level at Moscow University was quite low. When Pirogov reached the end of his studies, he had not yet participated in a single operation (the extraction of a tooth included) or even watched a cadaver dissection. Some of his teachers considered prayers and pilgrimages as suitable treatments for various diseases [4].

However, two teachers inspired Pirogov at Moscow University—Professor Loder and Professor Mudrov. Loder encouraged Pirogov to study anatomy seriously. Professor Mudrov taught his students to treat not only the illness but the whole patient—an approach which remains till date in modern medicine [2].

In May 1828, Pirogov, then age 17, qualified as a physician. Professor Mukhin encouraged him to participate as a candidate in the postgraduate international Institute of the University of Dorpat (Tartu). Pirogov succeeded in passing the entry exams and gained a scholarship from the Russian government to begin his training in the Baltic-German University of Dorpat in July that same year. Only 20 Russian students were accepted to Dorpat every year [2]. In Dorpat, Pirogov mainly gained knowledge from books and not from experiments or observation [3]. Dorpat students were prepared to become lecturers in Russian universities. The Dorpat professors fixed a curriculum for each student according to his needs and abilities. Pirogov was chosen to study anatomy and surgery and worked under the supervision of Professor Wachter. Wachter taught him anatomy on fresh cadavers and specimens preserved in ethanol. In surgery, Pirogov was instructed by Professor Moier, a student of Antonio Scarpa. Pirogov lived in Moier’s house for a time, practically as a family member. Moier also supplemented Pirogov’s education with musical and literature interests [5]. Pirogov was inspired by Moier, and Moier was taken by Pirogov’s passion and dedication to the field of anatomy [4].

In 1829, Pirogov was released from some compulsory lectures to complete his doctoral thesis, “The feasibility of treating aneurysms at the inguinal artery by ligation of the abnormal aorta.” Pirogov applied his method to animals and proved the feasibility of his technique, which achieved a gradual obliteration at the aorta, avoiding paralysis of the hind limbs and pelvis. He then carried out the operation on patients with aneurysms of the inguinal artery, an operation in which Cooper had previously failed [6]. Pirogov completed his studies in Dorpat after defending his thesis on September 27, 1832, which was published in Germany shortly thereafter [2,7].

In May 1833, Pirogov visited Berlin to expand his knowledge of anatomy and surgery. At the Charite University Hospital, he met F. Schlemm, Professor of Anatomy, and Friedrich Dieffenbach, Professor of Surgery [2]. The famous surgeons Johann Rust and Carl Ferdinand von Graafe disappointed Pirogov. Dieffenbach, despite his excellent surgical skills in plastic surgery, also received criticism from Pirogov due to Dieffenbach’s complete ignorance of anatomy and physiology. In Berlin, Pirogov met a remarkable woman anatomist—Mrs. Vogelsang—who was an accomplished anatomist and provided Pirogov cadavers for his dissections [4]. Pirogov also met Conrad Langebeck in Germany, who was Professor of Surgery, Anatomy, and Ophthalmology at Gottingen University. Langebeck was the best of his tutors and inspired his students and Pirogov with his extensive knowledge in anatomy and surgery science [8].

In May 1845, Pirogov left Berlin to visit St. Petersburg, but during his journey, he got sick with typhus and stayed in Riga until that September [7]. After Pirogov heard the Chair of Surgery at Moscow University was taken by F.I. Inozentzev, he stayed in Dorpat as ordinary professor and director at the Surgical Clinic. In March 1836, Pirogov was appointed as a full professor of surgery at Dorpat University and as Moier’s successor [2]. Pirogov’s students at Dorpat University did not easily welcome him; he did not speak fluent German. Only a few weeks into the semester, however, Pirogov was well-respected and loved by his students [3]. Pirogov demanded that his students provide analytical explanations for every medical decision they made. Before every operation, he thoroughly reviewed with his students the anatomy of the region in which they were operating [5].

Pirogov had the strong belief that every physician should disclose every error made during his medical practice. He held that disclosing mistakes is important for preventing further errors and limiting consequences for a patient [3,9].

From 1836–1840, there were a large number of ophthalmologic cases in the Baltics. At that time, Pirogov and 15 medical students founded the first ophthalmologic clinic in the Baltics with 10 beds [5]. In addition to his surgical skills in several areas, Pirogov had a deep knowledge of artery trunks and fasciae. He studied the location of the fascia in relation to the neighboring blood vessels, muscles, and nerves. Moreover, he applied the Indian rhinoplastic method as modified by Dieffenbach. From 1836–1841, Pirogov supervised the doctorate theses of several students in Dorpat [2,5]. Moreover, Pirogov studied the healing of Achilles tendon wounds [5]. In 1836, he performed his first tenotomy on a 14-year-old girl with a club foot, a procedure considered therapeutically effective at the time. He discovered the tendon is surrounded by two sheaths, not one, as was previously believed [2]. In 1837, Pirogov introduced the use of a stiff bandage in clinical practice. In 1838, Nikolai visited France where he met the famous Professor of Anatomy and Surgery Velpeau and was impressed by him [5].

In 1341, Pirogov left Dorpat University for the position of chairman of the department of surgery at the Medical–Surgical Imperial Academy in St. Petersburg [1]. Despite the opposition he met, he managed to establish a hospital clinic in connection to the Academic Chair of Surgery [3]. The situation he found in St. Petersburg was dramatic. The Army Hospital provided poor hygiene conditions to the patients, with overcrowded rooms of 60 to 100 beds; and the patients suffered from hospital gangrene, erysipelas, and sepsis [4]. Pirogov faced difficulties apart from the hospital conditions in that he had enemies among the other professors at the academy. A local journalist was bribed to slander Pirogov, patients were paid to accuse Pirogov of malpractice, and there was even a plot to declare him insane [10]. Pirogov never took money from his patients; he was knowable, easy to find, and maintained a high sense of democracy [3]. It is also worth mentioning a little-known anecdote in which Pirogov helped a destitute family in Kiev financially and medically. The family could not afford food, and their infant was dying of a severe disease. The story of Pirogov’s help became known in the city of Kiev and was recorded by the famous Aleksandr Kuprin [11].

Razanowski summarized Pirogov’s time in St. Petersburg: “While he disserts a large hospital, he brings an anatomical institute into being and becomes its chief” while concurrently lecturing on topographical anatomy and pathology and consulting at several hospitals [12]. During his service as the Surgical Chair at St. Petersburg, Pirogov performed 11,000 dissections. In 1841, he began an analytical study in topographical anatomy. He made frozen sections of cadavers, taking advantage of the cold Russian winter. The result of his effort was the “Anatomia topographical sections per corps humanum congelatum triplice directione ductis illustrate,” consisting of four volumes with 224 illustrations published from 1852–1859 [4,13-14]. Notably, “Pirogov’s triangle” is the famous anatomical space in the neck he discovered whose superior boundary is formed by the hypoglossal nerve, the inferior boundary is formed by the intermediate tendon of the digastric muscle, and the posterior border is formed by the posterior border of the mylohyoid muscle [15].

Pirogov lost his first wife to meningitis after the birth of his second son. After this tragedy, he visited Italy, France, and Germany, and in 1847, he visited the Caucasus where he practiced advanced surgery and became the first surgeon to use etherization in Europe [3,16]. He was intimately aware of the extreme conditions and complications of war. For the first time, operations were performed without the screams of patients. The psychological influence of anesthesia on the soldiers was remarkable [17]. Pirogov developed his own inhaler device and the equipment for using ether rectally [18-19].

Pirogov, in his early 50s, decided to resign from his position at St. Petersburg Hospital. He could not tolerate the ignorance of most of the professors at the hospital and their lack of sincere interest in patient health. Anesthesia, surgical instruments, and an evidence-based medical approach were considered luxury assets, given the hospital’s finances [10].

During the Crimean War campaign at Sevastopol in 1854, Pirogov accepted the position of surgeon general from the grand duchess Elena Pavlovna. Pirogov and the duchess had conceived of the idea of sending female nurses to treat wounded and sick soldiers (a first in Europe), building the foundation for the later establishment of the Russian Red Cross [3,20]. The nurses were divided into three groups: surgical assistants, housekeepers, and apothecaries. They were extremely helpful during the patients’ hospitalization, in both military hospital administration and the confrontation of corruption [10].

In the battlefields of the Crimean War, Pirogov encountered dramatic situations. He and his assistants performed several hundred operations daily. Pirogov and his female nurses and physicians fell ill with typhus. Patients were positioned on boards, one next to the other, with no differentiation of their wounds—clean and unclean wounds were treated in the same way. The transportation of the patients was completely inadequate, and the supplies of linen and medical instruments were grossly insufficient [3].

Moreover, Pirogov encountered administrative corruption in the military hospitals, and he quickly realized that the patients primarily suffered from factors due to insufficient administration. The epidemics and medical mistakes were only secondary causes of suffering for the patient soldiers. Pirogov’s priority was to uncover bad management and corruption in the military medical administration [10,21].

During the Crimean War, Pirogov conceived of the first “triage” system for the wounded soldiers. The battle casualties were distributed and isolated as quickly as possible. The seriously injured were moved into hospitals, away from the battlefront. Surgical operations were never carried out during battles; rather, patients were evacuated to military hospitals. The injured patients were classified into four categories: the hopeless who were relegated to the priest and nurses, those who needed operations urgently, those whose operations could be postponed for one or two days and, therefore, were evacuated to a nearby hospital, and the slightly wounded who were able to walk and were, therefore, sent to the nearest hospital to be treated and then returned to their units [4,22-23].

Pirogov introduced an amputation method known as Pirogov’s amputation in 1854, which consists of the amputation of the foot through the distal part of the tibia and fibula with a retention of part of the calcaneus. This leaves an excellent weight-bearing condition without prosthesis [24]. Despite his own method, Pirogov opposed the strong opinions of the time that stated amputation is preferred to conservative treatment. He was convinced that indiscriminate amputations were carried out in vain. He amputated only when a fixed plaster-of-Paris cast could not be applied [10,22].

Pirogov was ultimately disappointed in the military hierarchy system and resigned from military medicine. The Minister of Education offered him the position of the Curator of Education for the Odessa District. For five years, he oversaw the selection of professors in Russians Universities [25-27]. As the Curator of Education, Pirogov traveled extensively to improve the educational system. After his retirement, he lived for five years, traveling between Berlin and Heidelberg [27].

In 1881, Pirogov was diagnosed with a space-occupying lesion in his mouth. Moscow’s professors concluded the lesion was malignant. An operation was deemed essential and urgently, but when he was examined by the famous Billroth, Pirogov was told his disease was not serious, and it would pass without any surgical intervention. Pirogov remained at his estate and died later that year, on November 25, 1881. Billroth later stated that Pirogov would not have been able to withstand the operation, and the lesion would recur anyhow [3].

## Conclusions

Although Pirogov lived 200 years ago, his life and work are still worth mentioning. His scientific means of thinking, his humanistic mentality, and his democratic beliefs are an example for modern physicians and an inspiration for researchers. Pirogov’s penetrating point of view will always rank him among the greatest in the history of medicine.
